# Alcoholism and *Strongyloides stercoralis*: Daily Ethanol Ingestion Has a Positive Correlation with the Frequency of *Strongyloides* Larvae in the Stools

**DOI:** 10.1371/journal.pntd.0000717

**Published:** 2010-06-22

**Authors:** Carla Couzi Marques, Maria da Penha Zago-Gomes, Carlos Sandoval Gonçalves, Fausto Edmundo Lima Pereira

**Affiliations:** 1 Serviço de Gastroenterologia do Hospital Universitário C A Moraes, Vitória, Espírito Santo, Brazil; 2 Núcleo de Doenças Infecciosas do Centro de Ciências da Saúde, UFES, Vitória, Espírito Santo, Brazil; National Institute of Parasitic Diseases, China

## Abstract

**Background:**

Significantly higher prevalence of *Strongyloides stercoralis* has been reported in chronic alcoholic patients. The aim of this investigation was to report the prevalence of *Strongyloides* larvae in stools of chronic alcoholic patients with known daily ethanol intake.

**Methods:**

From January 2001 through December 2003 the results of fecal examinations and the daily ethanol intake were retrieved from the records of 263 chronic alcoholic and from 590 non-alcoholic male patients that sought health care at the outpatients unit of the University Hospital C A Moraes. Alcoholic patients were separated into four groups, with 150g intervals between the groups according to the daily ethanol intake.

**Results:**

(a) The frequency of *Strongyloides* was significantly higher in alcoholic patients than in control group (overall prevalence in alcoholic 20.5% versus 4.4% in control group; p = 0.001). Even in the group with a daily intake of ethanol equal to or less than 150g the prevalence was higher than in control group, although non significant (9.5%, versus 4.4% in control group; p = 0,071); (b) the prevalence of *Strongyloides* in alcoholic patients rises with the increase of ethanol intake (Pearson's Correlation Coefficient = 0.956; p = 0.022), even in patients without liver cirrhosis (Pearson's Correlation Coefficient = 0.927; p = 0.037).

**Conclusion:**

These results confirm and reinforce the hypothesis that chronic alcoholism is associated with *Strongyloides* infection, which is in direct relationship with the severity of alcoholism, independently of the presence of liver cirrhosis.

## Author Summary

It has been reported that *Strongyloides stercoralis* infection is more prevalent in chronic alcoholic patients than in non alcoholics living in the same country. In a retrospective study on the prevalence of *S. stercoralis* infection in a large sample of alcoholic patients, we demonstrate that this prevalence is significantly higher than in non-alcoholic patients admitted at the same hospital. Moreover, the frequency of the parasite was in close relationship with the daily amount of ingested ethanol, even in the absence of liver cirrhosis, reinforcing the idea that chronic alcoholism is associated with increased susceptibility to *Strongyloides* infection. Beside the bad hygiene profile of alcoholic patients, which explains high risk for acquisition of the parasite, the high prevalence of *S. stercoralis* in alcoholics may be in relationship with other effects of ethanol on the intestinal motility, steroid metabolism and immune system, which could enhance the chance of autoinfection and the survival and fecundity of females in duodenum. In this way, the number of larvae in the stools is higher in alcoholic patients, increasing the chance of a positive result in a stool examination by sedimentation method.

## Introduction

The presence of *Strongyloides stercoralis* larvae was reported to be higher in the stools of chronic alcoholics than in the stools of nonalcoholic patients at the same hospital in two different studies in Brazil (in Uberlândia, MG [1] and in Vitória, ES [2]).

The association of ethanol abuse and *Strongyloides* also has been reported by other researchers, although control groups were not used in these observations. In a report on the prevalence of *Strongyloides* in patients with liver cirrhosis in Juiz de Fora, MG, Brazil [3], the data presented showed a high prevalence of the larvae in patients with liver cirrhosis associated with chronic alcoholism. However, the authors did not comment on the possible effect of ethanol abuse on *Strongyloides* infection. Avendaño et al [4] found 5% of *Strongyloides* in stools of 106 chronic alcoholics in Costa Rica. These authors did not use controls. However the prevalence observed was higher than the 0.9% reported in a national survey for intestinal parasites, although in this survey a less sensitive method to identify *Strongyloides* larvae was used. Adedayo et al [5] reported 27 cases of disseminated strongyloidiasis in the Caribbean island of Dominica, and found that chronic alcoholism was a relevant factor associated with poor prognosis of infection. Recently, Silva et al. [6], while describing the presence of *Strongyloides* infection in HIV/AIDS patients, emphasized that the prevalence was significantly higher in HIV/AIDS patients with abusive use of ethanol than in non alcoholic HIV/AIDS patients.

Higher prevalence of *Strongyloides* has been reported in alcoholic patients with liver cirrhosis and chronic pancreatitis than in alcoholic patients without these complications of alcoholism [1]. Although this observation has not been confirmed in another study with a large sample of chronic alcoholic patients [2], high prevalence of the parasite in cirrhotic is an indirect demonstration of a correlation between the severity of alcoholism and the prevalence of *Strongyloides* in alcoholic patients. However great number of heavy drinkers never develops liver cirrhosis or chronic pancreatitis and, in all the reports cited above, there was no attempt to establish a relationship between the severity of alcoholism and the prevalence of *Strongyloides*. It could be said that the higher the ethanol intake the higher the alterations in the defense mechanisms would be, which could promote the installation and survival of *Strongyloides* larvae. To test this hypothesis we investigated the frequency of *Strongyloides* larvae in the stools of chronic alcoholics, whose daily ethanol intake was also evaluated.

## Methods

Records of 563 alcoholic patients at the outpatient integrated health care unit for alcoholic patients at the University Hospital C A Moraes from January 2001 through December 2003, were reviewed. The inclusion criteria were: adult male, in which records there was result of fecal examination and sure information on presence or absence of liver cirrhosis and on daily ethanol intake. After reviewing all records, 263 chronic alcoholic patients (103 with liver cirrhosis and 160 without cirrhosis) were included in the study.

The results of stool examinations of 590 non alcoholic male patients, admitted to the same hospital, during the same period, were collected from the records and used as a general prevalence of *Strongyloides* in non-alcoholic patients that received medical care at this hospital. Records of patients who attended to different divisions of the Hospital, and who fulfilled the following inclusion criteria, were selected: adult male, 20–65 years old, in which record there was a result of fecal examination and information necessary to exclude chronic alcoholism.

For all patients, alcoholic and non alcoholic, the stool examinations were carried out in three samples using the sedimentation method.

The diagnosis of chronic alcoholism was according to the WHO criteria (F10.2, ICD 10, 2002).Presence of liver cirrhosis was confirmed by clinical, laboratory, image and endoscopy for esophageal varices.

To calculate the correlation between the ethanol intake and the prevalence of *Strongyloides* larvae in stools, the chronic alcoholic patients were divided into four groups, according to their daily ethanol intake: 60 to 150g/day, 151 to 300g/day, 301 to 450g/day,and over 450g/day.

Statistical calculations were carried out using the version 9.0 of SPSS for Windows Frequencies were calculated with 95% confidence intervals and the Pearson correlation test was used to correlate the frequency of larvae in stool examinations with the daily ethanol ingestion. To calculate the Pearson's correlation coefficient it was considered as a reference value for daily ethanol ingestion for each group the highest value for each class interval: 150g (group 60–150g), 300g (group 151–300g), 450g (group 301–450g) and 600g ( group over 451g). Values of p less than 0.05 were considered significant.

This research was approved by the Ethical Committee of the Federal University of Espirito Santo. All patients signed a consent form allowing the use of data registered in their records for the present research objective.

## Results

The mean age was 46.5±9.1 years (median 44) in the 263 alcoholic patients and 44.8±16.1years (median 42) in the non alcoholic group. We did not collect sure information on socioeconomic pattern of each alcoholic and non alcoholic patient. The great majority of patients (86%) in both groups came from the neighborhoods of the urban periphery of metropolitan Vitória where people with low income live in similar sanitary conditions.

Table 1 summarizes the prevalence of *Strongyloides stercoralis* in stools of the chronic alcoholics according to the daily intake of ethanol and the frequency of *Strongyloides* larvae in stools of the non alcoholic group.

**Table 1:** Frequency of *Strongyloides* larvae in stools of 263 chronic alcoholic patients grouped according the daily ethanol intake and in 590 nonalcoholic patients admitted at the same hospital.

Daily ethanol intake (g)	*S.stercoralis*		% (95% CI)	P value*
	Positive	Negative		
Non alcoholic patients (N = 590)	26	564	4.4 (2.8–6.0)	-
60–150 (N = 53)	5	48	9.5 (2.3–16.7)	0.071
151–300 (N = 91)	13	78	14.3 (7.2–21.5)	<0.001
301–450 (N = 92)	24	68	26,1 (17.2–35.0)	<0.001
>450 (N = 27)	12	15	48.0 (29.2–66.8)	<0.001
All alcoholic patients (N = 263)	54	209	20.5 (15.7–25.3)	<0.001

*Chi-square or Fisher exact test in comparison with non alcoholic group.

The overall frequency of *Strongyloides* in chronic alcoholics was 20.5% (95% CI = 15.7–25.3), significantly higher than 4.4% (95% CI =  2.8–6.1) in non alcoholic patients admitted to the same hospital. Other nematodes identified were, respectively in alcoholic and non alcoholic group: *Ascaris lumbricoides* ( 6.7%×5.4%), *Trichiuris trichiura* (1.5%×1.3%), Ancilostomidae (1,14%×0.8%), *Schistosoma mansoni* (0.25%×1.0%) and *Taenia* sp (0.02%×0.015%). No significant differences between the frequencies were observed. Cirrhosis was diagnosed in 103 alcoholic patients. In this group the prevalence of *Strongyloides* was 17% versus 23% in alcoholic patients without cirrhosis (p = 0,842).

The analysis of the frequency of *Strongyloides* larvae in stools of the different groups of alcoholic patients, separated by the daily ethanol intake, demonstrated a strong correlation between these two variables (Pearson's correlation coefficient = 0.956; p = 0.022). In addition, Chi square comparison demonstrated significant differences in the frequency of *Strongyloides* among the different groups of alcoholic patients (Chi square = 22.54; p<0.001) , and between each group of alcoholic patients and the non alcoholic group, except for the group with ethanol ingestion 60 to 150g/day, as shown in Table 1. When analyzed the correlation between the amount of daily ethanol intake and the prevalence of *Strongyloides* in the group without cirrhosis (160 patients) a significant correlation is maintained (Pearson's correlation coefficient = 0.927, p = 0.037).

## Discussion

The samples analyzed included only male alcoholics, because chronic alcoholism is more frequent in men. At the University Hospital outpatient unit for integrated health care for alcoholics, the male to female ratio for alcoholic patients is around 11∶1 [7].

Although the sedimentation method is not highly sensitivity to *Strongyloides* larvae identification, it was used in both alcoholic and control groups, allowing an impartial comparison.

The results presented here confirm that the prevalence of *Strongyloides* larvae in stools of chronic alcoholic patients is higher than in non alcoholic patients admitted to the same hospital. Prevalence of other helminthes did not differ between alcoholic and nonalcoholic patients, confirming our previous report [2]. Although we did not collect accurate information on socioeconomic pattern of patients, samples were collected from patients coming from similar neighborhoods (low-income dwellings), where they live in similar sanitary conditions. Thus the conclusion that the prevalence of *Strongyloides* larvae is higher in alcoholic than in non-alcoholic group may consider the caveats resultant from the assumption that the two samples are comparable. As we reported earlier [2] there was not a significant difference between the frequencies of *S stercoralis* between patient with or without cirrhosis. In addition, the data demonstrated that there is a positive correlation between the intensity of alcoholism and the prevalence of the parasite in stools and that this positive correlation persists if we consider only the patients without cirrhosis. This observation corroborates and reinforces the hypothesis that chronic alcoholism favors the infection of *Strongyloides stercoralis* and/or enhances the survival of the worm in the duodenum and/or enhances larval elimination, thus increasing the chance of a positive stool examination. The worm infection and/or survival or increased larval elimination is favored even in absence of liver cirrhosis.

Although the observations presented here confirm and reinforce the idea that chronic ethanol intake is a factor associated with *Strongyloides* infection, there is not a clear-cut explanation for the high frequency of this parasite in alcoholics. We do not know if the high prevalence of *Strongyloides* in chronic alcoholics is due to low hygiene profiles, favoring the infection or reduction in intestinal motility, favoring autoinfection or immune deregulation induced by ethanol, favoring parasite survival and autoinfection.

The reduction of gastrointestinal transit caused by effects of ethanol on muscle proteins or on vagal stimulation [8], [9] may be a factor enhancing autoinfection. Decreased intestinal motility permits delay of rhabditiform larvae in intestinal lumen, favoring the chance of their maturing to the infective filariform larvae, thus increasing the risk of autoinfection [10].

Oliveira et al. [1] and Zago-Gomes et al. [2] emphasize immune deregulation as the main factor favoring parasite survival and autoinfection. In fact chronic ethanol abuse interferes with the innate and adaptive immune response, both in human beings and experimental animals (review in [11]–[16]). There are defects in T cell functions, especially in helper T cell type 1 (TH1) immune response [17]. However, it seems that helper T cell type 2 (TH2) immune response is not impaired by the consumption of ethanol [18], [19]. Mice chronically treated with ethanol presented normal response to larvae of *S. stercoralis* inoculated into chambers implanted in the subcutaneous tissue [20]. For these reasons it is difficult to pin point immunosuppression induced by ethanol as the major factor for the increased prevalence of *Strongyloides* in chronic alcoholics, because the TH2 response, the most important resistance against this worm in humans [21], [22], is not depressed by ethanol abuse. However, cells of the innate immune system may have functional impairment in chronic alcoholics (review in [13]–[16]) and some of these cells as neutrophils and eosinophils, play a role in innate and adaptive immune response to *Strongyloides* larvae, as demonstrated in mice [23], [24].

Other mechanisms triggered by ethanol abuse may be involved in the development of rhabditiform larvae, increasing their differentiation into infective filariform larvae, thus favoring autoinfection. In this way, the number of females would be increased in the duodenum, increasing the number of rhabditiform larvae in the stools, enhancing their chance to be found in a stool examination. Two such mechanisms may be considered: (a) an increased production of steroid metabolites that resemble ecdysones [25] and (b) a deregulation of immune response leading to alterations in antibody or T cells functions, that can interfere with larval development, as demonstrated in experimental animals [26] and humans [27].

Ethanol influences steroid metabolism by interfering with the activity of hepatic microsome oxidases [28] and increasing the corticoid production by enhancing the activity of the hypothalamus-hypophysis-axis [29]. Both effects can increase the level of steroid metabolites and some of them are similar to ecdysones, which are hormones that regulate the development of the nematode larvae [25]. Thus the increased levels of corticoid metabolites could enhance the fecundity of female worms, increasing the number of rhabditiform larvae and the direct development of these larvae into the infective filariform larvae, favoring autoinfection.

Although ethanol abuse preserves the TH2 function, it is possible that the depressed TH1 function and reduction in innate immune mechanisms may interfere with immune mechanisms that influence the developmental choice of developing rhabditiform larvae. The possible influence of host immune response on larval development is supported by the evidence, in rats, that the absence of anti-*Strongyloides ratti* immune response favors the direct development of larvae into the infective filariform larvae, while the indirect development into free living male and female adults is favored in the presence of such immune response [26]. An apparent opposite effect of immune dysfunction seems to affect the development of *S. stercoralis* larvae, as demonstrated in HIV infected patients, super-infected with the nematode. An inverse relationship between the number of CD4+ circulating T cells and the direct development of rhabditiform larvae into infective filariform larvae was demonstrated in those patients [27], an indication that immune deregulation also interferes with the development of *S. stercoralis* larvae. Although the effect of host immune status on the development of *Strongyloides* is different between *S. ratti* and *S. stercoralis* in their respective hosts, it is possible that immune deregulation may influence the larval development. As chronic alcohol abuse interferes with the immune response, this immune deviation may be favoring the direct development of rhabditiform larvae into infective filariform larvae thus enhancing autoinfection and increasing the number of worms in the duodenum and the chance of finding larvae in the stool examination.

In conclusion our results indicate that ethanol abuse is a factor associated with the high prevalence of *Strongyloides* larvae in stools, with a positive correlation to the intensity of alcoholism, even in absence of liver cirrhosis. The association of chronic alcoholism with *Strongyloides* infection may be either by the direct effect of ethanol on the immune system, or by factors closely associated to living conditions of severely alcoholized persons. Further investigations are necessary to uncover the possible mechanisms involved in the increased number of *Strongyloides* larvae in the stools of chronic alcoholic patients.

## References

[1] Oliveira LCM, Ribeiro CT, Mendes DM, Oliveira TC, Costa-Cruz JM (2002). Frequency of *Strongyloides stercoralis* infection in alcoholics.. Mem Inst Oswaldo Cruz.

[2] Zago-Gomes MP, Aikawa KF, Perazzio SF, Goncalves CS, Pereira FE (2002). Prevalence of intestinal nematodes in alcoholic patients.. Rev Soc Brasil Med Trop.

[3] Gaburri D, Gaburri AK, Hubner E, Lopes MH, Ribeiro AM (1997). Intestinal parasitosis and hepatic cirrhosis.. Arq Gastroenterol.

[4] Avendaño L, Hernandez F, Jimenez F, Avila A, Castro D (1999). Strongyloides stercoralis em pacientes alcohólicos.. Parasitol Dia.

[5] Adedayo O, Grell G, Bellot P (2002). Hyperinfective strongyloidiasis in the medical ward: review of 27 cases in 5 years.. South Med J.

[6] Silva CV, Ferreira MS, Borges AS, Costa-Cruz JM (2005). Intestinal parasitic infections in HIV/AIDS patients: experience at a teaching hospital in central Brazil.. Scand J Infect Dis.

[7] Macieira MS, Zago-Gomes MP, Garcia MLT (1993). Programa de atendimento ao alcoolista do Hospital Universitário Cassiano Antônio de Moraes da Universidade Federal do Espírito Santo (PAA-HUCAM-UFES).. J Bras Psiq.

[8] Addolorato G, Montalto M, Capristo E, Certo M, Fedeli G (1997). Influence of alcohol on gastrointestinal motility: lactulose breath hydrogen testing in orocecal transit time in chronic alcoholics, social drinkers and teetotaler subjects.. Hepatogastroenterology.

[9] Wegener M, Schaffstein J, Dilger U, Coenen C, Wedmann B (1991). Gastrointestinal transit of solid-liquid meal in chronic alcoholics.. Dig Dis Sci.

[10] Marcial-Rojas R, Marcial-Rojas. R  (1971). Strongyloidiasis.. Pathology of Protozoal and Helminthic Diseases with Clinical Correlations..

[11] Mendenhall CL, Theus SA, Roselle GA, Grossman CJ, Rouster SD (1997). Biphasic in vivo immune function after low- versus high-dose alcohol consumption.. Alcohol.

[12] Cook RT (1998). Alcohol abuse, alcoholism, and damage to the immune system.. Alcohol Clin Exp Res.

[13] Szabo G (1999). Consequences of alcohol consumption on host defense.. Alcohol Alcohol.

[14] Diaz LE, Montero A, Gonzalez-Gross M, Vallejo AI, Romeo J (2002). Influence of alcohol consumption on immunological status: a review.. Eur J Clin Nutr.

[15] Brown LA, Cook RT, Jerrells TR, Kolls JK, Nagy LE (2006). Acute and chronic alcohol abuse modulate immunity.. Alcohol Clin Exp Res.

[16] Szabo G, Mandrekar PA (2009). A recent perspective on alcohol, immunity, and host defense.. Alcohol Clin Exp Res.

[17] Waltenbaugh C, Vasquez K, Peterson JD (1998). Alcohol consumption alters antigen-specific Th1 responses: mechanisms of deficit and repair.. Alcohol Clin Exp Res.

[18] Gonzalez-Quintela A, Vidal C, Lojo S, Perez LF, Otero-Anton E (1999). Serum cytokines and increased total serum IgE in alcoholics.. Ann Allergy Asthma Immunol.

[19] Dominguez-Santalla MJ, Vidal C, Vinuela J, Perez LF, Gonzalez-Quintela A (2001). Increased serum IgE in alcoholics: relationship with Th1/Th2 cytokine production by stimulated blood mononuclear cells.. Alcohol Clin Exp Res.

[20] Krolewiecki AJ, Leon S, Scott PA, Nolan TJ, Schad GA (2001). Effect of chronic ethanol consumption on protective T-helper 1 and T-helper 2 immune responses against the parasites *Leishmania major* and *Strongyloides stercoralis* in mice.. Alcohol Clin Exp Res.

[21] Neva FA, Filho JO, Gam AA, Thompson R, Freitas V (1998). Interferon-gamma and interleukin-4 responses in relation to serum IgE levels in persons infected with human T lymphotropic virus type I and Strongyloides stercoralis.. J Infect Dis.

[22] Porto AF, Neva FA, Bittencourt H, Lisboa W, Thompson R (2001). HTLV-1 decreases Th2 type of immune response in patients with strongyloidiasis.. Parasite Immunol.

[23] Padigel UM, Lee JJ, Nolan TJ, Schad GA, Abraham D (2006). Eosinophils can function as antigen-presenting cells to induce primary and secondary immune responses to Strongyloides stercoralis.. Infect Immun.

[24] Galioto AM, Hess JA, Nolan TJ, Schad GA, Lee JJ (2006). Role of eosinophils and neutrophils in innate and adaptive protective immunity to larval strongyloides stercoralis in mice.. Infect Immun.

[25] Genta RM (1992). Dysregulation of strongyloidiasis: a new hypothesis.. Clin Microbiol Rev.

[26] Harvey SC, Gemmill AW, Read AF, Viney ME (2000). The control of morpho development in the parasitic nematode *Strongyloides ratti*.. Proc Biol Sci.

[27] Viney ME, Brown M, Omoding NE, Bailey JW, Gardner MP (2004). Why does HIV infection not lead to disseminated strongyloidiasis?. J Inf Dis.

[28] Lieber CS (1997). Cytochrome P-4502E1: its physiological and pathological role.. Physiol Rev.

[29] Ogilvie K, Lee S, Weiss B, Rivier C (1998). Mechanisms mediating the influence of alcohol on the hypothalamic-pituitary-adrenal axis responses to immune and nonimmune signals.. Alcohol Clin Exp Research.

